# Investigations upon the Improvement of Dermatophyte Identification Using an Online Mass Spectrometry Application

**DOI:** 10.3390/jof8010073

**Published:** 2022-01-11

**Authors:** Arnaud Jabet, Anne-Cécile Normand, Alicia Moreno-Sabater, Jacques Guillot, Veronica Risco-Castillo, Sophie Brun, Magalie Demar, Romain Blaizot, Cécile Nabet, Ann Packeu, Renaud Piarroux

**Affiliations:** 1 AP-HP (Assistance Publique-Hôpitaux de Paris), Service de Parasitologie Mycologie, Hôpital La Pitié-Salpêtrière, 75013 Paris, France; arnaud.jabet@aphp.fr (A.J.); cecile.nabet@aphp.fr (C.N.); renaud.piarroux@aphp.fr (R.P.); 2 AP-HP, Hôpital Saint-Antoine, Service de Parasitologie Mycologie, Sorbonne Université, 75012 Paris, France; alicia.moreno-sabater@aphp.fr; 3 Inserm, Centre d’Immunologie et des Maladies Infectieuses (CIMI-Paris), AP-HP, Hôpital Pitié-Salpêtrière, 75013 Paris, France; 4 Dynamic Research Group, Ecole Nationale Vétérinaire d’Alfort, UPEC, USC ANSES, 94700 Maisons-Alfort, France; jacques.guillot@oniris-nantes.fr (J.G.); veronica.risco-castillo@vet-alfort.fr (V.R.-C.); 5 Department of Parasitology-Mycology, Ecole Nationale Vétérinaire, Agroalimentaire et de L’alimentation, Oniris, 44307 Nantes, France; 6 Service de Parasitologie-Mycologie, Ecole Nationale Vétérinaire d’Alfort, Biopole Alfort, 94700 Maisons-Alfort, France; 7 Centre Hospitalier Universitaire Vétérinaire de la Faune Sauvage (Chuv-FS), Ecole nationale vétérinaire d’Alfort, 94700 Maisons-Alfort, France; 8 AP-HP, Hôpital Avicenne, Service de Parasitologie-Mycologie, 93000 Bobigny, France; sophie.brun@aphp.fr; 9 EA3593 Ecosystèmes Amazoniens et Pathologie Tropicale, Université de Guyane, 97300 Cayenne, French Guiana; magalie.demar@ch-cayenne.fr (M.D.); romain.blaizot@ch-cayenne.fr (R.B.); 10 Hôpital Andrée Rosemon, Laboratoire Hospitalo-Universitaire de Parasitologie-Mycologie, 97300 Cayenne, French Guiana; 11 Service de Dermatologie, Cayenne Hospital, CEDEX 97300 Cayenne, French Guiana; 12 Inserm, Institut Pierre Louis d’Epidemiologie et de Santé Publique, Sorbonne Université, 75571 Paris, France; 13 Sciensano, BCCM/IHEM Collection, Mycology and Aerobiology Unit, 1000 Brussels, Belgium; ann.packeu@sciensano.be

**Keywords:** dermatophytes, MALDI-TOF, mass spectrometry, MSI-2

## Abstract

Online MALDI-TOF mass spectrometry applications, such as MSI-2, have been shown to help identify dermatophytes, but recurrent errors are still observed between phylogenetically close species. The objective of this study was to assess different approaches to reduce the occurrence of such errors by adding new reference spectra to the MSI-2 application. Nine libraries were set up, comprising an increasing number of spectra obtained from reference strains that were submitted to various culture durations on two distinct culture media: Sabouraud gentamicin chloramphenicol medium and IDFP Conidia medium. The final library included spectra from 111 strains of 20 species obtained from cultures on both media collected every three days after the appearance of the colony. The performance of each library was then analyzed using a cross-validation approach. The spectra acquisitions were carried out using a Microflex Bruker spectrometer. Diversifying the references and adding spectra from various culture media and culture durations improved identification performance. The percentage of correct identification at the species level rose from 63.4 to 91.7% when combining all approaches. Nevertheless, residual confusion between close species, such as *Trichophyton rubrum*, *Trichophyton violaceum* and *Trichophyton soudanense*, remained. To distinguish between these species, mass spectrometry identification should take into account basic morphological and/or clinico-epidemiological features.

## 1. Introduction

Dermatophytes are a monophyletic clade of keratinophilic fungi (*Arthrodermataceae*) that are frequent skin pathogens in human and animals [1,2]. Until the 2000 s, dermatophyte identification was mostly based on macroscopic and microscopic morphological features. This conventional approach requires well-trained professionals and extended culture time. MALDI-TOF mass spectrometry appeared as an alternative allowing earlier and standardized identification of fungal colonies. Many studies have been published since 2008 [3,4] demonstrating the interest of this identification method. The best performances have been obtained with “in-house” libraries and not commercial libraries [5]. Nevertheless, recurrent errors still occurred regarding closely related species such as *T*. *rubrum*, *T. violaceum* and *T. soudanense*; *Trichophyton interdigitale* and *Trichophyton mentagrophytes*; *Trichophyton equinum* and *Trichophyton tonsurans*; and *Microsporum audouinii* and *Microsporum canis* [5,6,7,8,9,10]. The boundaries between these species or even the validity of some of them have been controversial [11,12,13,14]. In 2017, the updated taxonomy of dermatophytes [1] gathered them in species complexes, calling for further studies to better distinguish species within these complexes. Recent studies on the *T. rubrum* complex [15,16] and *T.* *equinum/T. tonsurans* [17] complex have tended to confirm the validity of the species boundaries. In contrast, many genotypes have been described inside the *T.* *interdigitale/T. mentagrophytes* complex, raising new questions about the corresponding species boundaries [18]. Finally, *Microsporum* species distinction appeared less controversial [19,20].

To improve MALDI-TOF mass spectral fungal identification, an online application named MSI was launched in 2017. Even if it allowed for an improvement of dermatophyte identification, mistakes between closely related species were still observed with MSI [21,22,23,24]. Sharing most of its references with MSI, MSI-2 (https://msi.happy-dev.fr/ (accessed on 12 December 2021)) is a new application typified by more accurate algorithms and an extended fungal database [25]. The aim of this study was to improve MSI-2’s library of references for the identification of dermatophytes, investigating two improvements: increasing the number of reference strains per species and including more spectra per reference strain obtained from cultures of various ages. Furthermore, as a new culture plate named ID-fungi plate (IDFP) (Conidia, Lyon, France) has been developed to facilitate mass spectra realization from fungal cultures, we also investigated the interest of including spectra from cultures on this media in the MSI-2 references.

## 2. Materials and Methods

### 2.1. Strains and Culture

Fifty-five strains of dermatophytes were collected from four teaching hospitals (La Pitié-Salpêtrière and Saint-Antoine hospitals in Paris, France, Avicenne Hospital in Bobigny, France and Cayenne Hospital in French Guiana, France), twenty-eight from the national veterinary College of Alfort and twenty-eight additional strains from BCCM/IHEM Fungi Collection (Brussels, Belgium). The 111 strains used in this study are listed in Table S1. They correspond to 20 dermatophyte species (1 to 14 strains per species).

The strains were reseeded on two culture plates: Sabouraud medium with gentamicin and chloramphenicol but without cycloheximide (Oxoid, Dardilly, France) (SGC) and IDFP medium. The media were incubated at 25 °C until all successive mass spectra were obtained.

### 2.2. Sequencing

Sequencing of the ITS1-5.8S-ITS2 region was performed for all strains and was used as the reference for the identifications. The primers used were ITS1 (TCCGTAGGTGAACCTGCGG) and ITS4c (TCCTCCGCTTATTGATATGC). DNA extraction was performed on an Emag machine (Biomérieux, Marcy-l’Étoile, France). The reaction mixture for PCR consisted of 30 µL of 1X LC480 mix (Roche Diagnostics, Basel, Switzerland) with 0.3 µL of the primer pair at a concentration of 10 pmol/µL and 2 µL of DNA extract. The PCR program consisted of an initial 10 min denaturation step at 94 °C followed by 40 cycles of 20 s denaturation at 94 °C, 30 s annealing at 55 °C and 60 s elongation at 72 °C and finally a final elongation step of 7 min at 72 °C. Sequencing was carried out using an ABI3730 DNA sequencer (Thermo Fisher Scientific, Waltham, MA, USA) via the Sanger technique. Species identification was obtained through the alignment of the ITS sequences of the isolates with those of the reference strains [1] available in GenBank in MEGA X software version 10.1.8 (Kumar, Stecher, Li, Knyaz, and Tamura 2018) to compare the base-to-base sequences. Indeed, it has been demonstrated that database results by automated comparison may be inaccurate [26].

The genotype determination of isolates belonging to three complexes of closely related species (*T. rubrum* complex [15], *T. interdigitale*/*T. mentagrophytes* complex [18] and *T. tonsurans*/*T. equinum* [27] complex) was carried out via comparison with the ITS sequences of published strains also available in GenBank.

### 2.3. MALDI-TOF Mass Spectrometry

The cultures were subjected to the MALDI-TOF extraction protocol as soon as the growth of the dermatophyte was sufficient to allow the collection of biological material without fear of preventing the further growth of the fungus. For cultures on SGC plates, the mass spectra were acquired approximately every three days, at least until the twentieth day post-seeding, to obtain at least five series of successive spectra. For cultures on IDFP medium, mass spectra were acquired approximately every three days, at least until the tenth day post-seeding, to obtain at least three series of successive spectra. The acquired spectra were gathered in files according to the chronology of the realization of the spectra and the culture medium. Files S1 to S5 group together the five series of spectra obtained from isolates grown on SGC plates, and I1 to I3 group together the three series acquired from the same isolates grown on IDFP.

Mass spectra were acquired after complete protein extraction. A small fragment of colony was collected from the culture dish with a scalpel blade and suspended in a mixture of 300 µL of sterile water and 900 µL of anhydrous ethyl alcohol in a sterile 1.5 mL tube. After centrifugation for 2 min at 13,000× *g*, the pellet was resuspended in 10 µL of 70% formic acid for 5 min before the addition of 10 µL of 100% acetonitrile and incubation for 5 min. After further centrifugation (13,000× *g*, 2 min), 1 μL of supernatant was deposited in quadruplicate for the same extract on a polished steel target. After air drying, 1 µL of matrix (HCCA) was applied to each deposit. The spectra acquisitions were carried out using a Microflex Bruker spectrometer (Bruker Daltonics, Bremen, Germany), and the manufacturer’s default parameters were applied. In the event of an acquisition failure according to Bruker’s criteria, the obtained spectra were not rejected; instead, the sum of the rejected spectra was saved for each deposit in question.

### 2.4. Libraries of Spectra

The original spectra library (L0) that was included in MSI-2 before this study included 612 representative spectra of 51 different species with one to eight different strains per species (Table S2). We used L0 as a starting point to determine the impact of the addition of spectra from cultures of various ages and on two different media. For that purpose, nine new libraries were created. Libraries L1 to L5 were formed by the successive addition to L0 of files S1 to S5 (spectra of increasing age acquired from cultures on SGC). For instance, L1 was formed by the addition to L0 of files S1 and L2 by the addition to L1 of file S2. Libraries L6 to L8 were formed on the same principle but by the addition to L0 of files I1 to I3 (spectra of increasing age acquired from cultures on IDFP). L9 was formed by the addition to L0 of all additional spectra acquired from cultures on SGC (S1 to S5) and IDFP (I1 to I3).

### 2.5. Cross-Validation

All spectra produced were submitted to each library. Self-recognitions of spectra obtained from the same strain were excluded. This indicates that a submitted spectrum could not be compared with a spectrum made from the same strain regardless of age or culture medium. Consequently, it was not possible to assess the results corresponding to the species represented by a single strain (*Arthroderma quadrifidum*, *Nannizzia incurvata* and *Paraphyton cookei*). Indeed, the prohibition of self-recognition makes these results irrelevant.

The identification threshold for MSI-2 was set at 20. A maximum of three identifications were returned, corresponding to the three species that gave the highest MSI-2 results. The first identification was compared with the result of ITS sequencing for each strain. If they were identical, the identification was considered correct; otherwise, it was considered incorrect. If the score was below 20, no identification was retained.

**Figure 1:**
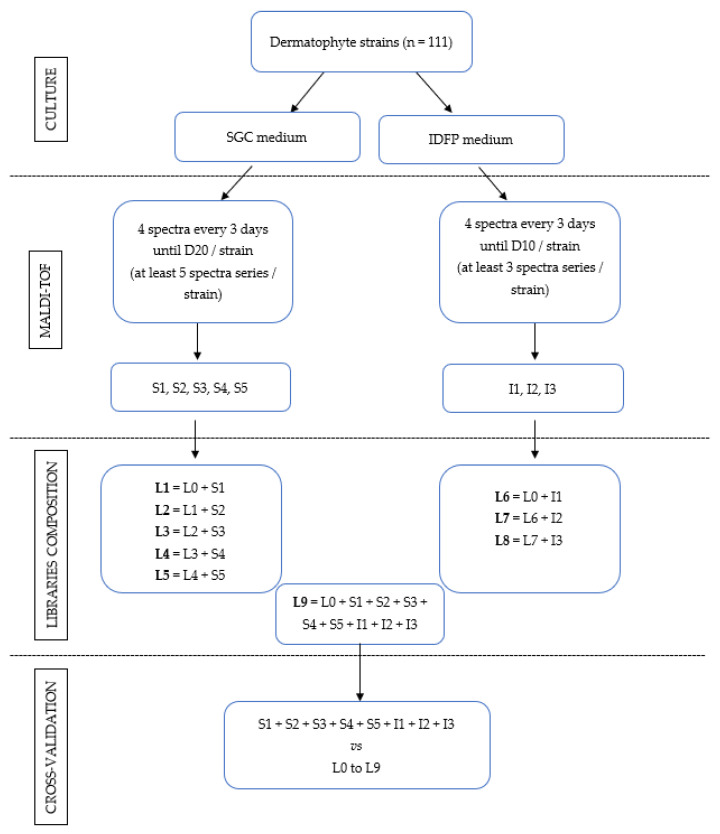
Overall design of the study. S1, S2, S3, S4 and S5 are files of spectra of increasing age acquired from cultures on Sabouraud medium plus gentamicin and chloramphenicol (SGC) medium. I1, I2 and I3 are files of spectra of increasing age acquired from cultures on ID-fungi plate (IDFP). L0 is constituted by spectra of BCCM/IHEM Fungi collection strains currently in MSI-2.

The global design of the study is summarized in Figure 1.

### 2.6. Statistical Analysis

Statistical analyses used McNemar’s nonparametric paired sample test and were performed using XLSTAT 2021.2.2software (Addinsoft, Paris, France), with α risk set at 5%.

## 3. Results

### 3.1. Overall Results

The overall results of the cross-validation are summarized in Table 1. A total of 3852 different spectra (including 2490 carried out on SGC and 1362 on IDFP) were taken into account for this comparison.

The gradual enrichment of the banks with spectra corresponding to late cultures on SGC was associated with an increase in the percentages of correct identification. The most marked gains concerned the passage from L0 to L1 (63.4% vs. 77.5%, *p* < 0.0001) and from L1 to L2 (77.5% vs. 85.9%, *p* < 0.0001), whereas lower but significant improvement was observed when comparing L2 to L5 (85.9% vs. 88.9%, *p* < 0.0001). Similar results were obtained with the libraries formed by the addition of spectra produced on the IDFP medium, with a significant progression between L0 and L6 (63.4% vs. 74.8%, *p* < 0.0001), L6 and L7 (74.8% vs. 84.4%, *p* < 0.0001) and L7 and L8 (84.3% vs. 85.8%, *p* < 0.001). The best results were obtained with L9, which integrates all new spectra carried out on SGC and IDFP, leading to an overall success rate of 91.7%, significantly better than the success rate of the L5 and L8 libraries. From L0 to L9, the identification gains were based both on a reduction in the number of errors (32.1% vs. 7.8%) and on the identification of previously unrecognized spectra (4.5% vs. 0.4%).

### 3.2. Results by Species and Species Complexes

When the results were studied by species, the impact of the addition of new spectra was variable (Table 2). For some species, the switch from L0 to L9 was associated with a marked improvement in the rates of correct identification, as was the case with *Epidermophyton floccosum* (56.7% vs. 100%), *Nannizzia fulva* (75.4% vs. 100%) or *Trichophyton benhamiae* (81.0% vs. 97.0%); conversely, there was no impact for *Nannizzia gypsea* or *Nannizzia persicolor*—the results were already excellent with L0, with 100 and 97.4% correct identification for these two species, respectively. Regarding *Trichophyton verrucosum*, the progress was major—no spectrum was recognized with L0—but the percentage of correct identification was not above 50.0% with L9. Regarding the complexes in which most errors occurred, the *T. rubrum* complex, *T. interdigitale*/*T. mentagrophytes*, *T. equinum*/*T. tonsurans* and *Microsporum* spp., the results differed according to the complex. For the *Microsporum* complex, with L9, 98.0% of the spectra were correctly identified at the species level (vs. 68.6% with L0), and there was only one remaining confusion between *M. audouinii* and *M. canis*. For the *T. rubrum* complex, the shift from L0 to L9 was associated with major progress in identifying *T. soudanense* (2.6% vs. 94.3%) and *T. violaceum* (19.6% vs. 80.4%) and 99.1% of the spectra were correctly identified at the complex level. However, at the species level, incorrect identifications still occurred in 9.2% of the cases. Regarding the *T. mentagrophytes*/*T. interdigitale* and *T. equinum*/*T. tonsurans* complexes, correct identification rates were 84.6% and 90.7% with L9, respectively, but incorrect identifications still occurred, not only between species inside the complexes but also between these two complexes (4.0 and 1.3% of the spectra, respectively, according to the complex). In this latter case, false recognitions corresponded to confusion between the “early” spectra of *T. interdigitale* and *T. equinum*. Within the *T. mentagrophytes*/*T. interdigitale* complex, incorrect identifications were unequally distributed among strains. A total of 48.7% of errors concerning the species *T. mentagrophytes* implied two strains corresponding to genotype II *: ENVA W985 and ROUT13, which were identified as *T. interdigitale*. Conversely, 53.1% of false identifications of *T. interdigitale* as *T. mentagrophytes* implied references from either of these two strains. Within the *T. equinum*/*T. tonsurans* complex, the IHEM13407 strain, representative of the ‘*T. tonsurans* DNA type 2’ genotype, was responsible for 38.6% of the errors, and 68.8% of its spectra were recognized as *T. equinum*.

### 3.3. Distinction of Correct and Incorrect Identifications

**Figure 2:**
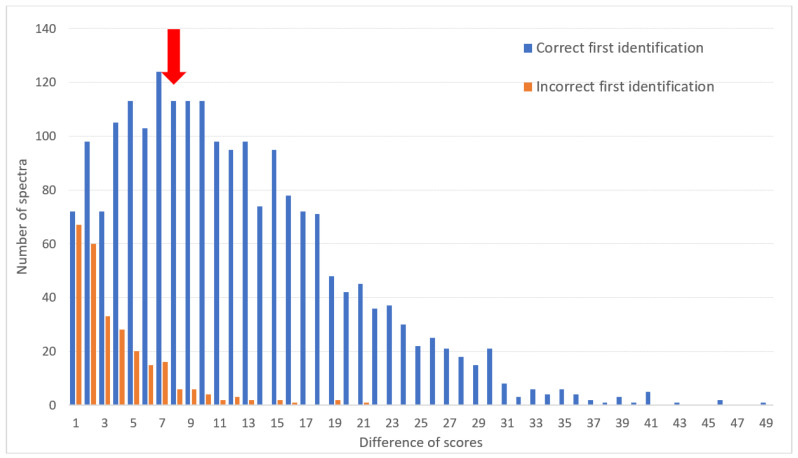
Difference between the score of the first identification and that of the second depending on whether the first identification was correct (results of identification with the L9 library and for the *T. rubrum* complex, *T. interdigitale/T. mentagrophytes* and *T. equinum/T. tonsurans* complexes). The red arrow indicates the chosen threshold (8) beyond which the identification result could be considered at the species level and below which it should be taken into account only at the complex level.

When considering both the first identification score and the second identification score given by MSI-2, it was possible to detect most identification errors observed within the *T. rubrum complex*, *T. interdigitale/T. mentagrophytes* and *T. equinum/T. tonsurans* complexes. Indeed, most false results corresponded to a small difference (i.e., less than 8) between the scores of the first and second species identified (Figure 2). Applying this threshold to take into account a species identification result would lead to 62.0% sensitivity and 91.4% specificity for correct identification at the species level. Below this threshold, the identifications could be at least considered at the complex level with a minimal chance of error of 3.5%.

## 4. Discussion

The aim of this work was to improve the MSI-2 library for dermatophyte identification. Thus, 3984 spectra were obtained from 111 molecularly identified strains, representative of 20 different species. They were acquired from cultures on two different media (SGC and IDFP) and at various ages of culture. The impact of adding these spectra to the current bank was assessed through a cross-validation experiment. The transition from the initial library (L0) to the library formed by the addition of all spectra produced (L9) was associated with a marked improvement in the overall rate of correct identification of the spectra to the species level (63.4% vs. 91.7%). The significant progression (63.4% vs. 77.5%) already obtained by the only addition of early spectra from cultures on SGC (L0 to L1) confirmed the major effect observed when multiplying reference strains of a given species [4]. The positive effect of the addition of late spectra (L1 compared to L4 and L5) and spectra from cultures on IDFP (L5 compared to L9) was also demonstrated. These results contradict some previous publications that only observed a limited impact of culture medium and age of colonies on the spectra, with minimal consequence on the accuracy of the identification [5,7].

Regarding the complexes in which incorrect identifications mostly occurred, major improvement was achieved for *Microsporum* spp. allowing distinction between *M. canis* and *M. audouinii* with high confidence. In contrast, for the *T. rubrum*, *T.* *interdigitale/T. mentagrophytes* and *T.* *equinum/T. tonsurans* complexes, confusion between closely related species still occurred. The difference between the scores of the first identified species and of the second species may help assess the accuracy of the identification. Nevertheless, basic morphological and/or clinico-epidemiological features should also be integrated in a polyphasic approach to improve the reliability and accuracy of identifications.

Thus, the location of the lesions (mostly *tinea pedis* and *tinea unguium* for *T. rubrum* and *tinea capitis* for *T. soudanense* and *T. violaceum*) and typical macroscopic morphological features (fluffy, white colonies for *T. rubrum*; small, glabrous, wrinkled, yellow or orange colonies for *T. soudanense*; or small, glabrous, wrinkled, purple colonies for *T. violaceum*) could be of interest to distinguish the species inside the *T. rubrum* complex. In addition to the well-known *T. rubrum*, *T. soudanense* and *T. violaceum* species, *T. kuryangei* was taken into account in this study. Indeed, according to recent publications [15,16], *T. kuryangei* constitutes a specific clade inside the *T. rubrum* complex. It has been implicated as an agent of *tinea capitis*, and colonies have been described as *T. rubrum*-like colonies. 

Regarding *T. equinum*/*T. tonsurans* differentiation, the epidemiological context of the infection (zoonotic infection involving horses or interhuman transmission) is a key element. In our study, a strain corresponding to ‘*T. tonsurans* DNA type 2’ (IHEM 13407) generated almost 40% of the misidentifications inside the *T. equinum*/*T. tonsurans* complex. This may indicate that this genotype is actually closer to *T. equinum* than to *T. tonsurans*. Suppressing the spectra of this strain could probably improve the performance of the application in distinguishing these two species.

*Trichophyton interdigitale* and *T. mentagrophytes* share similar morphological features. *Trichophyton interdigitale* is considered an anthropophilic species mostly involved in *tinea pedis* and *tinea unguium,* whereas *T. mentagrophytes* is a zoophilic species that causes inflammatory lesions of *tinea capitis* and *tinea corporis* in humans [1,18]. However, two genotypes (VII and VIII) challenge this dichotomy. They have been associated with *T. mentagrophytes* based on ITS sequencing, but their transmission is interhuman, and the lesions are mostly *tinea genitalis* or *tinea cruris* and *tinea corporis,* respectively [28,29]. It has recently been proposed to elevate *T. mentagrophytes* genotype VIII to the species level as *T. indotineae* [30,31]. Moreover, *T. mentagrophytes* genotype II * is molecularly closer to *T. interdigitale* genotypes than *T. mentagrophytes* based on ITS sequences [18]. This could explain why this genotype is more often implicated in species confusion than the others. Waiting for taxonomic clarifications and further MALDI-TOF studies, atypical clinical cases may benefit from ITS sequencing for genotype determination.

Our study has limitations. While the most common anthropophilic, zoophilic and geophilic species were included in our work, it is not, however, exhaustive. Some anthropophilic species, such as *T. schoenleinii*, *T. concentricum* and *M. ferrugineum,* as well as zoophilic species, such as *T. quinckeanum* or geophilic species, were not included in this study because they are exceptionally observed in developed countries [32]. For some species, the progress observed in our study is still insufficient, particularly for *T. verrucosum* or *T. violaceum*, and the addition of new references is therefore necessary. While many strains of *T. mentagrophytes* were included, our study only imperfectly reflects the diversity of genotypes grouped within the species. Genotypes VII and VIII in particular are not represented. There are also taxonomic uncertainties over the species *T. benhamiae*, which could be divided into four species [33]. The continual renewal of the taxonomy requires regular updating of the database.

## 5. Conclusions

This enriched library for dermatophyte identification may offer users improved performance and was conceived to be adapted to most laboratory practices (spectra of various ages and two culture media). Residual misidentifications are linked to molecular proximity between species, and dermatophyte PCRs can encounter the same kind of limitations [34]. The difference between the score of the first identified species and the score of the second one and/or basic morphological or clinico-epidemiological features could be integrated to obtain the most accurate identifications.

## Figures and Tables

**Table 1:** General results (%) of the cross-validation.

Libraries	L0	L1	L2	L3	L4	L5	L6	L7	L8	L9
		SGC Libraries					IDFP Libraries			
**Composition** **of the libraries**	MSI-2 references	L0 + S1	L1 + S2	L2 + S3	L3 + S4	L4 + S5	L0 + I1	L6 + I2	L7 + I3	L0 + S + I
**Correct identification**	63.4	77.5	85.9	86.9	87.6	88.9	74.8	84.3	85.8	91.7
**Incorrect identification**	32.1	20.8	13.2	12.4	11.8	10.5	22.7	14.4	13.3	7.8
**No identification**	4.5	1.7	0.9	0.8	0.6	0.6	2.5	1.2	0.9	0.5

The L0 library corresponds to the spectra from IHEM strains currently present in MSI-2. L1 to L5 libraries were formed by gradual addition to L0 of increasingly late spectra produced from isolates cultivated on Sabouraud medium plus gentamicin and chloramphenicol (SGC). L6 to L8 libraries were formed on the same principle from the same isolates cultivated on ID-fungi plate (IDFP). The L9 library was composed by adding all spectra acquired to L0.

**Table 2:** Percentages of correct identification by species with the L0 and L9 libraries.

Libraries	L0	L9
Composition of the Libraries	MSI-2 References	L0 + S + I
*Epidermophyton floccosum* (*n* = 120)	56.7	100
*M. audouinii* (*n* = 360)	57.2	96.9
*M. canis* (*n* = 236)	86.0	99.6
*N. fulva* (*n* = 65)	75.4	100
*N. gypsea* (*n* = 198)	100	100
*N. persicolor* (*n* = 76)	97.4	97.4
*T. benhamiae* (*n* = 100)	81.0	97.0
*T. equinum* (*n* = 258)	28.0	89.3
*T. erinacei* (*n* = 168)	96.9	100
*T. interdigitale* (*n* = 439)	63.6	81.8
*Trichophyton kuryangei* (*n* = 96)	62.5	77.1
*T. mentagrophytes* (*n* = 352)	71.6	88.1
*T. rubrum* (*n* = 454)	84.1	91.6
*T. soudanense* (*n* = 348)	2.6	94.3
*T. tonsurans* (*n* = 442)	60.2	91.2
*T. verrucosum* (*n* = 48)	0.0	50.0
*T. violaceum* (*n* = 92)	19.6	80.4

## Data Availability

Data (raw spectra and DNA sequences) can be obtained freely by contacting the corresponding author.

## Supplementary Materials

The following supporting information can be downloaded at https://www.mdpi.com/article/10.3390/jof8010073/s1. Table S1: Strains included in the study, corresponding genotype and number of associated spectra. Table S2: Composition of the L0 library.
